# Dynamic Driving Mechanism of Dual Structural Effects on the Correlation between Economic Growth and CO_2_ Emissions: Evidence from a Typical Transformation Region

**DOI:** 10.3390/ijerph19073970

**Published:** 2022-03-26

**Authors:** Yu Cai, Haiyan Duan, Zhiqiang Luo, Zhiyuan Duan, Xian’en Wang

**Affiliations:** 1 College of New Energy and Environment, Jilin University, Changchun 130012, China; cai.yu@sljg.mee.gov.cn (Y.C.); wxen@jlu.edu.cn (X.W.); 2 Songliao River Basin Ecology and Environment Administration, Ministry of Ecology and Environment, Changchun 130021, China; 3 Energy and Food Integrated Administrative Enforcement Detachment of Jinan City, Jinan 250000, China; mathilde9007@163.com

**Keywords:** driving mechanism, CO_2_ emissions, economic growth, dual structural effects, marginal utility, transformation region

## Abstract

How will the dual structural effects, represented by industrial structure and energy structure, affect the future correlation between economic growth and CO_2_ emissions? Taking Jilin Province as an example, this study explores the dynamic driving mechanism of dual structural effects on the correlation between economic growth and CO_2_ emissions by innovatively building an integrated simulation model from 1995 to 2015 and setting different scenarios from 2016 to 2050. Correspondingly, the concept of marginal utility and the method of variance decomposition analysis are introduced to reveal the mechanism. The results show that the energy structure is different while the industrial structure tends to be similar when CO_2_ emissions reach the peak under different scenarios. The slower the dual structure adjustment, the more significant the upward trend appears before the peak. The contribution of the dual structural effects to CO_2_ emissions caused by unit GDP growth is basically the same in peak year. With the transformation of socio-economy, the positive driving effect of the industrial structure will gradually weaken, while the negative driving effect of the energy structure will gradually increase. The methods and results presented can provide insights into sensible trade-offs of CO_2_ emissions and economic growth in different countries/regions during structural transitions.

## 1. Introduction

While the development of industrialization and urbanization has promoted the development of economic globalization, it has also exacerbated the climate change crisis caused by the increase in CO_2_ emissions that stem largely from energy consumption. In the global context of tackling climate change, how to effectively suppress CO_2_ emissions while promoting economic growth has become a challenge that most countries are facing in the development process. The Paris Agreement, which entered into force in 2016, is a legally binding global emission reduction agreement that plans the global climate governance pattern after 2020. Different countries and regions have accordingly proposed specific action plans for CO_2_ emission reduction based on the principle of common but differentiated responsibilities, in order to achieve the pursuit of economic development and emission reduction synergistically.

In recent years, research on the relationship between economic growth and CO_2_ emissions has been increasing drastically. In terms of research methods, the related literature can be classified into four categories. The first category explores the contribution of the influencing factors of CO_2_ emissions including economic scale through decomposition models. Since the logarithmic mean Divisia index (LMDI) method has the advantages of path independence and provides perfect decomposition results without residual terms [1], it has been widely adopted in the decomposition analysis regarding CO_2_ emissions, and most studies have demonstrated that economic growth is the main contributor to the increase in CO_2_ emissions. For example, Ma et al. [2] took advantage of the LMDI method to quantify the driving factors of CO_2_ emissions in China from 2005 to 2016, showing that rapid economic growth is the most important reason for the increase in CO_2_ emissions. Du et al. [3] identified the drivers of changes in energy-related CO_2_ emissions of high-energy intensive industries in China during 1986–2013 based on the LMDI method, verifying that the expansion of economic scale was the leading force explaining why CO_2_ emissions increased. In addition, Dong et al. [4] and Wang et al. [5] also confirmed that economic output was the dominantly positive driving factor of CO_2_ emissions by using the LMDI model.

The second category is the quantitative or qualitative analysis of the impacts of the factors including economic growth on CO_2_ emissions through econometrics and statistical models. Quantitative analysis models mainly reflect the influence degree and influence direction through fitting model coefficients, including the stochastic impacts by regression on population, affluence and technology (STIRPAT) model [3,6,7], the vector autoregressive (VAR) model [8,9], the autoregressive distributed lag (ARDL) model [10,11], the generalized method of moments (GMM) model [12,13], etc. For example, Li et al. [6] used the STIRPAT model to investigate the effect of the rationalization and upgrading of manufacturing structure on CO_2_ emissions in China, and the simulation coefficients show that the greater the ratio of industrial output to GDP, the weaker the restricting effect of resource dependence on the emissions reduction of manufacturing structure. The qualitative analysis models such as the vector error correction model (VECM) and Granger causality analysis mainly explain the relationship between economic growth and CO_2_ emissions by judging the causal relationship. Jian et al. [14] applied VECM to investigate the long-term equilibrium and short-term causality relationship among influencing factors and CO_2_ emissions, and the results show the long-term cointegration relationship between them. Mirza et al. [15] used VECM to explore the existence of Grangers’ long run, short run and strong causalities between economic growth, energy consumption and CO_2_ emissions for Pakistan.

The third category investigates the relationship between economic growth and CO_2_ emissions based on the EKC model. Compared with other methods that qualitatively determine the causality between variables or quantitatively determine the degree of impact by calculating model coefficients, the EKC proposed by Grossman and Krueger can better reflect the dynamic relationship between economic growth and CO_2_ emissions at different stages of economic development [16]. The results of some studies conform to the EKC hypothesis characterized by an inverted U-shape [5,17,18], while others do not, showing an N-shaped [19,20,21] or even M-shaped, curve [22]. In general, the reasons leading to this phenomenon lie in three aspects. The first reason is the difference in the study objects and corresponding time series data. Most of these studies showed that the relationship between economic growth and CO_2_ emissions in underdeveloped regions does not meet the EKC hypothesis compared with developed regions [23,24]. Besides, specific time periods parallel specific socioeconomic conditions, leading to different results even for the same research object [5,8]. The second reason stems from whether gross domestic product (GDP), as an independent variable, is quadratic or cubic when building the model [25,26]. The last reason derives mainly from different methods for estimating the coefficients of the model, among which FMOLS, DOLS and ARDL have been most commonly used [27,28,29].

The fourth category detects the decoupling relationship between economic growth and CO_2_ emissions based on the Tapio decoupling model. The determination of the decoupling relationship is achieved by calculating the decoupling elasticity, which can be specifically expressed as strong decoupling, weak decoupling, expansive coupling, negative decoupling, strong negative decoupling and so on [30]. Wu et al. [31] conducted a Tapio decoupling analysis of economic growth and CO_2_ emissions with reference to 30 Chinese provinces from 2001 to 2015, finding that there is a strong decoupling relation between GDP and CO_2_ emissions. Taking Beijing and Shanghai from 2005 to 2015 as examples, Wang et al. [32] used decoupling analysis to explore the relationship between sectoral economic output and carbon emissions. Both cities experienced weak decoupling in construction, expansive negative decoupling in transport and expansive coupling in trade.

In terms of research object, it can be basically divided into developed or developing countries or regions from the perspective of development level. Among them, the discussions with regard to developed countries have not been frequently seen, and most of the related studies aim to verify that economic growth and CO_2_ emissions are in line with the EKC hypothesis [33,34]. Conversely, more empirical studies with developing countries as targets, especially China, have been extensively conducted. In terms of research content, it involves sector perspectives such as construction sector [35,36], transportation sector [37], manufacture sector [38], etc., or sub-region perspectives such as Beijing [39,40], Shanghai [41] and other provincial regions.

There are two deficiencies in the reviewed studies. The first one is the lack of explanation of what causes the dynamic changes in the correlation between economic growth and CO_2_ emissions. As discussed above, the majority of the studies only quantified the impact of economic growth as one of the influencing factors on CO_2_ emissions, or analyzed the correlation between economic growth on CO_2_ emissions from the perspective of EKC hypothesis and decoupling status. Very few studies explored what factors drive the formation of the correlation, especially with a focus on the dynamic impacts of structural effects on the correlation. As mentioned by Grossman and Krueger [42], structural effects, including the effects of both industrial structure and energy structure, have important impacts on economic growth and CO_2_ emissions. They are indispensable factors to conduct a comprehensive analysis of the driving mechanism of CO_2_ emissions.

The second deficiency is the lack of research on the traditional industrial regions, which refer to those whose industrial structure is dominated by traditional industrial sectors (such as steel, machinery or electricity). These regions are currently undergoing industrialization, but in a desperate need for transformation in the pattern of economic development from the originally traditional industrial mode to a modern development mode. The structural characteristics of such regions are generally reflected in two aspects. The industrial structure has begun to transit from high-energy-consuming and high-emitting traditional industries toward service and emerging industries, but the main driving force of economic development is still traditional industries. The energy structure has begun to transit from coal to oil, gas and renewable energy, but it is still dominated by coal consumption, with relatively lower energy efficiency. Many countries and regions in the world have experienced this transformation stage, such as the Ruhr area in Germany, the central part of the United Kingdom, and the Great Lakes area in the northeast of the United States. More developing regions are now undergoing such a stage. On the one hand, different from developed regions, such regions generally face the dual pressures of CO_2_ emission reduction while promoting high-quality economic growth. On the other hand, CO_2_ emission reduction practices in such regions play a critical role in reducing global CO_2_ emissions due to their large emission reduction potential.

Therefore, there are some questions that have not yet been thoroughly discussed in previous studies, especially for the large number of traditional industrial regions experiencing transformation in the world represented by China, that is, how will the dual structural effects, represented by industrial structure and energy structure, affect the future correlation between economic growth and CO_2_ emissions? To what extent should industrial structure and energy structure be adjusted to make CO_2_ emissions decouple from GDP growth? In-depth research on these issues is conducive to providing a path reference for carbon emission reduction in these regions while ensuring stable economic development.

To fill the gaps, this study selects Jilin Province, a typical transformation region in the Northeast China as the empirical target. First, we built an integrated simulation model based on the evolution of various factors from 1995 to 2015. The indicator CO_2_ emission intensity (CEI) (CO_2_ emissions per unit GDP) functions as a bridge connecting economic growth and CO_2_ emissions, on which basis we decomposed CEI into multiple factors through the Kaya identity and transformed the simulation model to contain only structural effects by the dynamic ordinary least squares (DOLS) method. Secondly, we simulated the correlation between economic growth and CO_2_ emissions, and the peaking pathways of CO_2_ emissions in four different scenarios were set according to different development patterns from 2016 to 2050. Finally, through introducing the concepts of marginal utility and total utility, we took advantage of variance decomposition analysis (VDA) based on the VAR model to explore the impacts of dual structural effects on the correlation between economic growth and CO_2_ emissions. The methods and results of the study are expected to provide reference for coordinating economic growth and CO_2_ emissions in the underdeveloped countries/regions undergoing structural transformation, especially in the context of CO_2_ emission reduction globally.

## 2. Methods

### 2.1. Integrated Simulation Model

**Figure 1:**
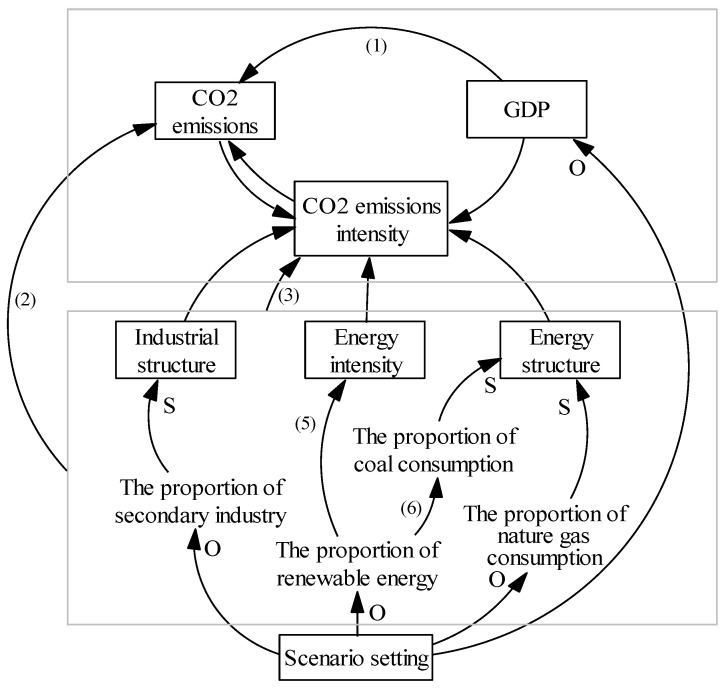
Integrated simulation modelling framework. (The numbers in the figure are consistent with the corresponding equations in the main context. Arrows without letters represent quantitative relationships; arrows with “S” represent representational relationships; arrows with “O” represent coverage relationships.)

In this study, an integrated simulation model is built based on the interaction among the socioeconomic, industrial, energy and emission variables, as shown in Figure 1.

This study uses a quadratic expression derived from the EKC modelling framework to analyze the correlation between economic growth represented by *GDP* and *CO_2_* emissions [16], as expressed in Equation (1).

(1) $$\ln CO_{2}=\alpha_{0}+\alpha_{1}\ln GDP_{it}+\alpha_{2}\ln GDP_{it}^{2}+\lambda_{it}$$

The prediction of *CO_2_* emissions is innovatively based on the combination of *GDP* and *CEI.* The prediction of the latter is based on the Kaya identity [43], which is built incorporating the influencing factors including industrial structure (*IS*) and energy structure (ES). By expanding the Kaya identity, *CO_2_* emissions can be expressed as Equation (2).

(2) $$CO_{2}=\sum_{ij}CO_{2,}{}_{ij}=\sum_{ij}GDP\frac{GDP_{i}}{GDP}\frac{E_{i}}{GDP_{i}}\frac{E_{ij}}{E_{i}}\frac{CO_{2,}{}_{ij}}{E_{ij}}=\sum_{ij}GDP\cdot IS_{i}\cdot EI_{i}\cdot ES_{ij}\cdot f_{ij}$$

where *E* denotes total energy consumption, *i* denotes the type of industry (primary industry, secondary industry or tertiary industry), *j* denotes the type of fossil energy (coal, oil or natural gas), *IS* denotes industrial structure of a region (the proportion of an industry’s added value in *GDP*), *FEI* denotes fossil energy intensity of an industry (fossil energy consumed by unit added value of an industry), *ES* denotes energy structure of an industry (the proportion of a type of fossil energy in total energy consumption), and ***f*** denotes *CO_2_* emission coefficient (*CO_2_* emissions of unit fossil energy consumption).

By transforming Equation (2), *CEI* can be expressed as:

(3) $$CI=\frac{CO_{2}}{GDP}=\sum_{ij}\frac{GDP_{i}}{GDP}\frac{E_{i}}{GDP_{i}}\frac{E_{ij}}{E_{i}}\frac{CO_{2,}{}_{ij}}{E_{ij}}=\sum_{ij}IS_{i}\cdot EI_{i}\cdot M_{ij}\cdot U_{ij}$$

According to Equation (3), *CEI* is closely related to industrial structure, *FEI* and energy structure. Since the primary industry represented by agriculture may remain stable without dramatic changes in the long run according to the national policy, the proportion of secondary industry’s added value in GDP (*SI*) is used to characterize industrial structure. The proportions of coal consumption (*CC*) and natural gas consumption (*NG*) in total energy consumption are used to characterize energy structure. Equation (3) can be extended after the logarithmization processing as Equation (4):

(4) lnCI=αlnSI+blnEI+clnCC+dlnNG+e

Based on the assumption that the use of renewable energy will effectively reduce *FEI* and coal consumption, the relationship between the proportion of renewable energy (*RN*) and *FEI* or *CC* is expressed as Equations (5) and (6), based on which Equation (4) can be further expressed as Equation (7).

(5) $$\ln EI=m\ln RN+\delta_{1}$$

(6) $$\ln CC=n\ln RN+\delta_{2}$$

(7) $$\ln CI=\alpha^{\prime }\ln SI+b^{\prime }\ln RN+c^{\prime }\ln NG+\lambda$$

where *a, b, c, d, m, n, a’, b’* and *c’* are fitting parameters, and *e, δ_1_, δ_2_* and *λ* are error terms.

Attributed to the capability of eliminating the endogeneity in regression and the sequence correlation in error terms [44], the dynamic ordinary least squares (DOLS) method is used for parameter estimation.

### 2.2. Marginal Utility, Total Utility and Variance Decomposition Analysis

In order to quantitatively analyze the impacts of structural effects on the correlation between GDP and CO_2_ emissions, the concepts of marginal utility (*MU*) and total utility (*TU*) in economics are introduced [45], as shown in Equation (8).

(8) MU=f(TU)=ΔTU/ΔQ

**Figure 2:**
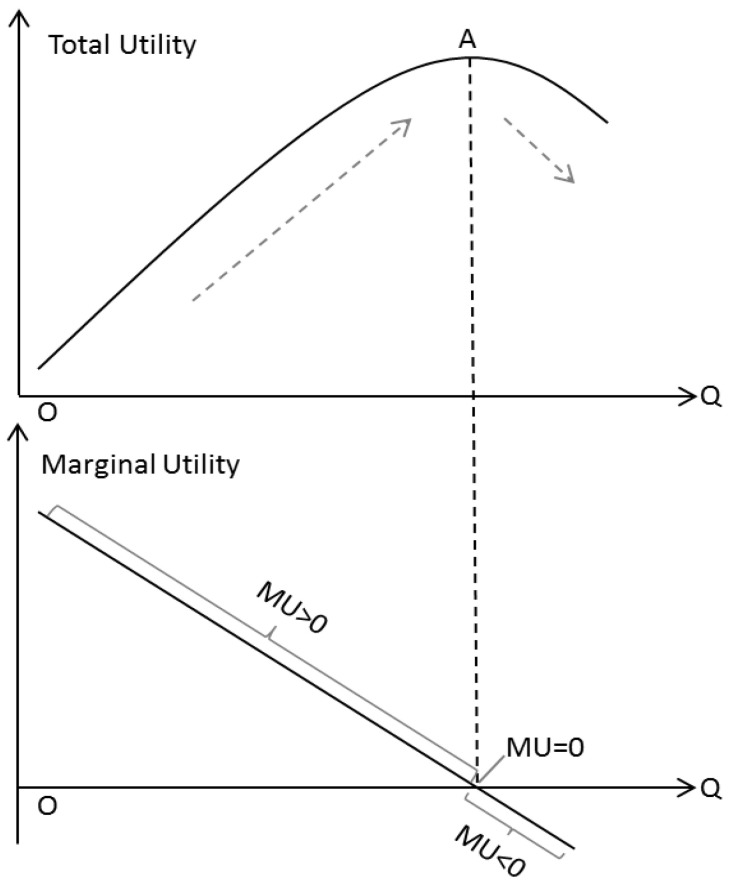
Marginal utility and total utility.

In the field of economics, *MU* refers to the degree of satisfaction per unit consumption of an item, *Q* refers to the amount of consumption and *TU* refers to total degree of satisfaction from the consumption of a certain number of items. Generally speaking, *MU* follows the law of gradual decrease, meaning that the first unit of consumption of a good or service yields more utility than the second and subsequent units, with a continuous reduction for greater amounts of consumption [46]. As illustrated in Figure 2, *MU* goes from positive to 0, and then to negative. Correspondingly, when *MU* > 0, *TU* goes up. When *MU* = 0, *TU* reaches the maximum value, that is, the turning point. When *MU* < 0, *TU* goes down.

Due to the similarity with Equation (1) in the inverted U-shaped trend, *TU* can be used to reflect the trend in CO_2_ emissions in the process of economic development. “CO_2_ emissions caused by unit GDP growth” (denoted by CUG, the same below) herein can be analogized to *MU*.

Based on this, the exploration of the influence of structural effects on the correlation between GDP and CO_2_ emissions reveals the contributions of industrial structure and energy structure to the changes in CUG, a perspective that has rarely been discussed before. We use VDA, which is based on the VAR model in econometrics, to quantify the contributions. The VAR model is an unstructured model containing multiple equations to estimate the dynamic relationship between all endogenous variables [8]. The principle is to take each endogenous variable in the system as the lag value of all endogenous variables, so as to extend the single variable autoregressive model to a VAR model composed of multivariate time series variables, as shown in Equations (9) and (10).

(9) $$Y_{t}=A_{0}+A_{1}Y_{t-1}+\cdot \cdot \cdot +A_{p}Y_{t-p}+\delta_{t}$$

(10) $$Y_{t}=\{\ln CO_{2,}{}_{t},\ln IS_{t},\ln RN_{t},\ln NG_{t}\}$$

where *Y_t_* is the time series of each variable; *A_0_,* *A_1_,* *…,* *A_p_* are *n* order coefficient matrix; *δ_t_* is the error term.

The premise of the stability of the VAR model is that the inverse roots of the AR characteristic polynomial is less than 1. On this basis, VDA can be carried out. VDA further evaluates the importance of different influencing factors by analyzing the contribution of each factor to the changes in endogenous variables. Therefore, VDA provides information about the relative importance of each random disturbance that affects the variables in the VAR model, which can explain the contribution of each influencing factor to the change of the dependent variables. This method can effectively explore the contribution of structural effects to changes in CUG. EViews 7.2 was employed to simulate the relevant results.

## 3. Data and Scenario Settings

### 3.1. Study Area

Jilin Province, located in the Northeast China, is a typical traditional industrial region. In the process of China’s industrial development, as the base of heavy industry, Jilin made a significant contribution to the country’s industrialization from the 1950s to the 1970s. However, with the upgrading of China’s industrial structure and the acceleration of modernization, Jilin has gradually been falling behind the eastern coastal regions. In addition, the adjustment of industrial structure and energy structure in Jilin has been sluggish in recent years. Meanwhile, the economic development level of Jilin has dropped below the national average level in recent years, ranking out of the top 20 in 31 provincial regions of China with even negative economic growth in 2019. What is more, Jilin has been listed as one of China’s 16 relatively high-carbon regions since 2010. Under the double pressures of economic growth and CO_2_ emissions reduction, the government has also proposed relevant plans for structural adjustment.

### 3.2. Data Source

This study employs the panel data of Jilin Province for the period 1995–2015 for parameter fitting. The data on GDP, fossil fuels consumption and the proportion of renewable energy come from the Jilin Province Statistical Yearbook [47]. The amount of CO_2_ emissions is calculated according to the National Greenhouse Gas Inventories published in the IPCC Guidelines [48], combined with the data on fossil fuel consumption. The descriptive statistics of the variables are presented in Table 1.

### 3.3. Scenario Setting

Scenario setting allows for adjusting the current development direction in a targeted manner based on foreseeable future development paths. In this study, we set four scenarios namely S1, S2, S3 and S4, each of which represented a different future development pattern. There were obvious hierarchical differences among four scenarios. Specifically, the settings for S1–S4 referred to the historical development trend of Jilin, the future development plan of Jilin, the average development level of China and the average development level of developed countries, respectively. The scenario setting was finally reflected in the parameter setting of the influencing factors involving *SI*, *RN*, *NG* and *GDP*, as shown in Table 2.

Among them, *SI* and *RN* were set based on the historical trends and related local plan [49] and national plan [50]. *GDP* was projected based on the historical trends and national planning, with the annual growth rate decreasing by 0.5% every five years [51]. Two reasons account for why the same parameter was set for *NG* in the four scenarios. On the one hand, *NG* in Jilin is very small, and the annual change is not significant, only increasing from 1% in 1995 to 3% in 2015. On the other hand, the future planning for *NG* in Jilin is not very optimistic, reaching a maximum of 18% by 2050 [52], far lower than the expectation for *RN*. Considering the limitations of natural gas development and to highlight the contribution of renewable energy, the variable control method was used to fix *NG*. By combining the settings for the three variables (*NG*, *IS* and *RN*), the results of *CEI* during 2016–2050 in the four scenarios can be obtained. The details of the above prediction results are displayed in Table 3.

## 4. Results

### 4.1. Correlation between Economic Growth and CO_2_ Emissions under Dual Structural Effects

In order to ensure the stability and accuracy of the estimation results, we conducted an Augmented Dickey–Fuller (ADF) unit root test and a Johansen co-integration test on the time series variables for 1995–2015. The results demonstrate that the stationarity requirements of the time series variables could be met and a long-term stable equilibrium relationship between the variables exists. The parameter estimation results for Equations (4)–(6) are presented in Table 4. The t-statistic follows a normal distribution, and the corresponding coefficient is significant at the confidence level of 1%. Based on this, the coefficients *α*’, *b*’, *c*’ and the error term *λ* in Equation (7) that only includes the representation of industrial structure and energy structure can be finally obtained as 0.91, −0.62, −0.15 and −1.84, respectively. 

Based on the quantitative relationships among the variables, we inputted the parameters set in the four scenarios into the integrated simulation model and estimated the corresponding CO_2_ emissions. Combining GDP set in the scenarios, we analyzed the correlation between GDP and CO_2_ emissions from 2016 to 2050 based on Equation (1) by using DOLS. The results of the parameters are presented in Table 5.

**Figure 3:**
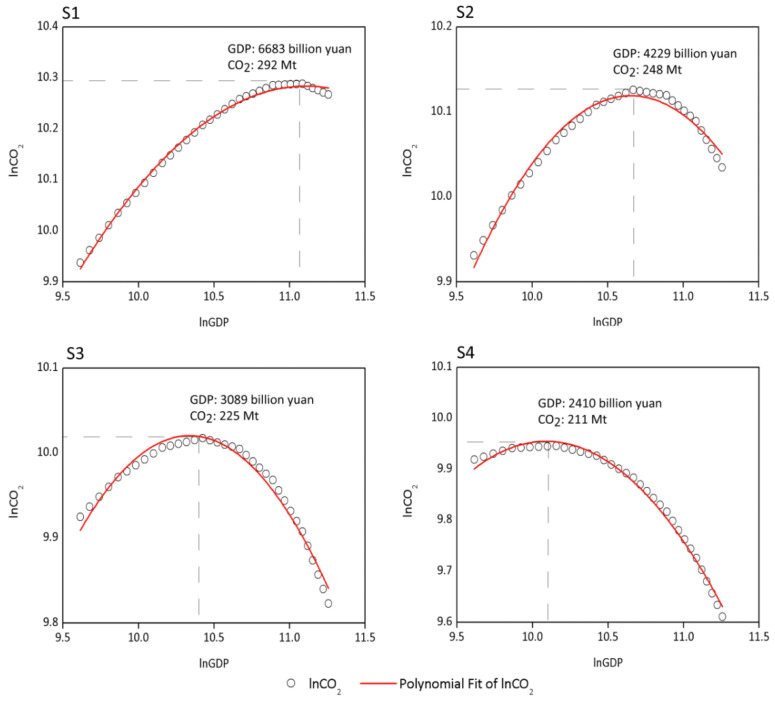
The correlation between GDP and CO_2_ emissions during 2016–2050 in four scenarios.

We used the framework of the EKC model to quantify the relationship between GDP and CO_2_ emissions, as it more intuitively shows the dynamic nonlinear relationship between two variables, which is more conducive to analyzing the impacts of structural effects along with the change of the relationship. The four scenarios reflect different evolution pathways of the correlation between GDP and CO_2_ emissions. As depicted in Figure 3, there is a turning point in the scenario, which means that GDP and CO_2_ emissions can be decoupled, that is, along with GDP growth, CO_2_ emissions can change from increasing to decreasing. However, it can be observed that the state at which the turning point appears in each scenario is significantly different. The faster GDP grows, the larger CO_2_ emissions, and the later the corresponding turning point appears. Specifically, in S1, the GDP at the turning point is 6683 billion yuan, with 292 Mt CO_2_ emissions correspondingly. The CO_2_ emissions slightly decline after reaching the turning point. Different changing trends in CO_2_ emissions after the turning point appear, reflecting different levels of decoupling between GDP and CO_2_ emissions. The GDP in S2, S3 and S4 at the turning point is 4229, 3089 and 2410 billion yuan, respectively, with corresponding CO_2_ emissions amounting to 248, 225 and 211 Mt, respectively. 

The different results at the turning points are induced by different levels of the influencing factors. The results of *CEI* and corresponding *NG*, *RN*, *SI*, *CC* and *FEI* at the turning point are displayed in Table 6. The CEI in the four scenarios is 4.51, 5.81, 6.65 and 8.09 t/thousand yuan, respectively. Natural gas accounts for 14.29%, 8.94%, 7.07% and 5.59% in total energy consumption. Renewable energy accounts for 22.98%, 16.60%, 13.90% and 11.21% in total energy consumption. There are no insignificant changes in industrial structure in the four scenarios, with *SI* ranging within 40–42%. It can be observed from the states of the variables at the turning point that *NG*, *RN* and *CC* all decrease from S1 to S4. The CEI and FEI increase from S1 to S4. Therefore, S1 is a better scenario in terms of a higher proportion of clean energy and lower FEI and CO_2_ emission intensity. However, if CO_2_ emissions are combined within the whole economic development process (see Figure 3), the scenarios cannot be simply judged according to the states of the variables, as the four turning points appear at different economic development stages. The turning point in S1 appears in 2043, while in 2035, 2029 and 2025 in S2, S3 and S4, respectively. Accordingly, structural adjustment and economic development are in S1 for a longer time compared with other scenarios. In terms of the impacts of structural effects on the changing trend in the correlation between GDP and CO_2_ emissions, the slower the adjustment of energy structure and industrial structure is, the more significant the growth trend in GDP will be before the turning point, resulting in significant differences in GDP and CO_2_ emissions at the turning point. Specifically, in the four scenarios, the annual average growth rate of *RN* before the turning point is 4.3%, 4.8%, 5.2% and 5.6%, while the average annual growth rate of *SI* is -0.6%, −1%, −1.4%, and −1.8%. Correspondingly, the difference in GDP between the base year and turning point-year is 5180, 2731, 1596 and 919 billion yuan, and the changes in CO_2_ emissions are 84, 42, 21 and 9 Mt. 

### 4.2. CO_2_ Emission Peak under Dual Structural Effects

**Figure 4:**
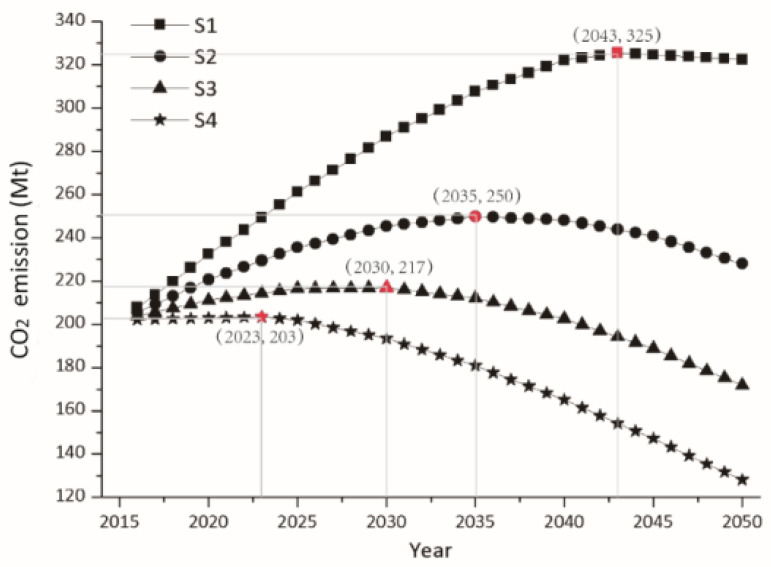
CO_2_ emissions trend and emissions peak in different scenarios from 2016 to 2050.

In addition to exploring the correlation between GDP and CO_2_ emissions, considering the importance of CO_2_ emission peak for absolute emission reductions, the impacts of structural effects in peaking CO_2_ emissions were also analyzed. The trends in CO_2_ emission in different scenarios from 2016 to 2050 were delineated in Figure 4. It can be found that the peak of CO_2_ emissions is also a turning point, but it is different from the turning point of EKC. The turning point of EKC represents the decoupling of GDP and CO_2_ emissions, that is, CUG begins to decline, which means a relative reduction in CO_2_ emissions, while the peak turning point means the absolute reduction in CO_2_ emissions. Specifically, the peaking time of CO_2_ emissions in S1–S4 is 2043, 2035, 2030 and 2023, respectively, with corresponding peaking amount as 325, 250, 217, and 203 Mt. Compared with S1, the peaking time in S2–S4 is 8, 13 and 20 years earlier, with corresponding peaking amount 75, 108 and 122 Mt smaller, respectively. The scale effect represented by GDP and the structural effects characterized by industrial structure and energy structure collectively determine the pathways towards CO_2_ emission peak. When CO_2_ emissions in the four scenarios reach the peak, the corresponding GDP is 6685, 4301, 3260 and 2241 billion yuan; *SI* is 42.09%, 40.75%, 40.32% and 43.08%; *RN* is 34.14%, 25.54%, 20.98% and 15.14%, respectively.

There is a positive correlation between GDP and CO_2_ emissions before the peak, showing an increasing trend, with the opposite conditions after reaching the turning point. When GDP growth rate is greater than the decrease rate in CEI, CO_2_ emissions show an upward trend; when GDP growth rate is smaller than the decrease rate of CEI, CO_2_ emissions show a downward trend. Therefore, in order to reach peak CO_2_ emissions as soon as possible, it is necessary to promote the decrease in CEI while controlling the economic growth rate to some extent. The dual structural effects characterized by industrial structure and energy structure are the main factors affecting CEI. Specifically, the increase in *RN* and *NG* and the decrease in *SI* are conducive to the decrease in CEI, thereby promoting the advancement of the peaking time and the reduction in the peaking emission amount.

### 4.3. Contributions of Structural Effects to the Correlation between Economic Growth and CO_2_ Emissions

According to the above analysis, the EKC model can effectively explore the non-linear relationship between GDP and CO_2_ emissions, but cannot clarify in detail how structural effects drive the evolution of the correlation between GDP and CO_2_ emissions. Therefore, we further introduced the concepts of TU and MU to deal with this. Referring to the principle of MU, we employed VDA based on the VAR model to quantify the dynamic impacts of structural effects on CUG. 

The contributions of CIS and CES to CUG in the four scenarios are illustrated in Table 7. It can be observed that the change in industrial structure (CIS) has a positive effect on CUG and the change in energy structure (CES) a negative effect on CUG. The average contribution of CES becomes enhanced (e.g., −5.56%, −6.48%, −7.69% and −8.56% during 2016–2020 and −9.34%, −11.01%, −12.93% and −14.32% during 2046–2050). The average contribution of CIS becomes weakened (e.g., 38.44%, 37.43%, 36.67% and 35.64% during 2016–2020 and 30.46%, 29.03%, 27.31% and 25.38% during 2046–2050). Industrialization is an important reason for the increase in CO_2_ emissions while promoting economic growth. With the optimization of the industrial structure, traditional industries characterized by high energy consumption and high emissions will gradually be replaced by emerging industries characterized by low carbon. Therefore, the positive contribution of CIS on CUG gradually weakened. In this process, the replacement of fossil energy by clean energy also directly led to a significant decline in the growth rate of CO_2_ emissions. The higher the proportion of clean energy, the more significant the negative contribution of CES to CUG.

When the turning point appears, the overall average contributions of CIS and CES are 23.79%, 24.81%, 23.11% and 23.63%, respectively, in the four scenarios. Although the turning points corresponding to different development patterns occur at different times, the contributions of structural effects are similar in the four scenarios (around 23–24%). This verifies that the decoupling between CO_2_ emissions and GDP can be achieved only when the collective impacts of industrial structure and energy structure have been adjusted to a certain level.

(1) When the decoupling between GDP and CO_2_ emissions has not occurred, this means that CO_2_ emissions will continue to increase with GDP growth. However, there are two different states at this stage. One is that CO_2_ emissions are accelerating with GDP growth, indicating that the traditional development mode is still continuing. The other is that with further GDP growth, the increased rate of CO_2_ emissions is gradually decreasing, indicating that structural optimization has begun. However, this optimization only results in relative reductions in CO_2_ emissions and has not yet resulted in absolute reductions, which is a typical performance in the early stage of transformation. (2) When decoupling has occurred, this indicates that industrial structure and energy structure have been adjusted to a certain extent. At this time, it is approximately in the middle stage of transformation, where the GDP growth rate is slowing down, the tertiary industry is replacing the secondary industry to occupy a dominant position and the share of coal consumption in total energy consumption is declining. (3) With further decoupling of GDP and CO_2_ emissions, the transformation region will eventually enter the late stage of transformation. Different from the previous states, under adequate support of technologies and investment, the space for the optimization of energy structure can be larger than that for industrial structure. Although the contribution of CIS to CUG is higher than that of CES during 2016–2050, this situation may be reversed. As can be observed from Table 7, the contribution of CIS to CUG in the four scenarios changes by 20.76%, 22.44%, 25.53% and 28.79% from 2016 to 2050, while that of CES changes by 67.99%, 69.91%, 68.14% and 67.29%. It can be concluded that CES has greater potential for CO_2_ emission reduction in the future.

## 5. Discussion and Policy Implications

Jilin Province is a typical transformation region implementing a dual adjustment of both industrial structure and energy structure. In this study, an integrated simulation model is built based on the interactions among the socioeconomic, industrial, energy and CO_2_ emission variables in Jilin from 1995 to 2015, using CEI as a bridge connecting GDP and CO_2_ emissions. There is an inverted U-shaped dynamic correlation between GDP and CO_2_ emissions. The turning points appearing in the four scenarios indicate that CO_2_ emissions can decouple from GDP in Jilin once it has undergone sufficient development. However, the states at the turning points in the four scenarios differ significantly. The slower the adjustment of energy structure and industrial structure is, the more significant the upward trend appears before the turning point; furthermore, the slower the decrease in CEI is, the later CO_2_ emissions decouple from GDP.

The turning point of the correlation between economic growth and CO_2_ emissions reflects the beginning of relative emissions reduction, while the CO_2_ emission peak is the beginning of absolute emissions reduction. The reduction in CO_2_ emission intensity is the main representation of relative emissions reduction, which is reflected in the gradual slowdown of the increase in CO_2_ emissions before the emission peak. GDP drives the increase in CO_2_ emissions, while CEI suppresses the increase in CO_2_ emissions. Only when the reduction rate of CEI is greater than GDP growth rate can CO_2_ emissions change from rising to falling, thereby forming the peak. Lower GDP growth rate and greater decrease in CEI caused by more aggressive adjustment of energy structure and industrial structure results in an earlier arrival of the emission peak, which is similar to the findings of Du et al. [35] and Shuai et al. [53].

In order to explore the dynamic influence mechanism of structural effects on the correlation between GDP and CO_2_ emissions, CO_2_ emissions and CUG were analogized to TU and MU in the process of economic development. CIS and CES have opposite effects on CUG with different intensities of influence. When the turning point occurs, *SI* in the four scenarios is similar (about 41%), while *RN* in the four scenarios is distinct (37%, 26%, 21% and 17%, respectively). Even if the levels of GDP and CO_2_ emissions in the four scenarios are different, also with differences in energy structure, the industrial structure tends to be similar, which is also in line with the socio-economic development characteristics of developed countries that have peaked CO_2_ emissions in their peak years [54]. With regard to the contribution to CUG, industrial structure and energy structure each makes similar contributions in the four scenarios. With the development of emerging industries and the optimization of energy utilization models, the positive contribution of CIS on CUG will gradually weaken while the negative contribution of CES to CUG will gradually increase. Although the turning points corresponding to different scenarios occur at different times, the contributions of structural effects to CUG are almost similar, which shows that structural adjustment plays a relatively fixed and irreplaceable role in the process of peaking CO_2_ emissions.

Based on the findings, the following policy implications are proposed. As a traditional industrial region that is undergoing transformation in terms of both industrial structure and energy structure, Jilin should strive to coordinate the relationship between economic growth and CO_2_ emission reduction. CEI is an important link to measure the relationship between economic growth and CO_2_ emissions. Currently, the CEI in Jilin Province is relatively high, which still has a large potential for emission reduction. Structural adjustment is the key to realizing the transformation of economic development mode in Jilin Province.

The adjustment of industrial structure is the prerequisite for development. As one of the typical representatives of the heavy industrial base, under the impact of emerging industries such as information technology at home and abroad, Jilin has gradually lost its previous development advantages. The industrial mode characterized by high energy consumption, high emissions and low added value cannot provide an impetus for the sustainable development of economy and society. At the same time, due to the limitations of geographical location and resource endowment, emerging industries and service industries with high added value and low energy consumption have not been effectively developed, which causes the lag of regional development [55]. Therefore, Jilin Province should reduce energy-intensive industries while eliminating backward production capacity, and vigorously develop technology-intensive and capital-intensive industries.

The adjustment of energy structure is the guarantee of industrial transformation. In accordance with the characteristics of high energy consumption and high emissions of traditional industries, fossil energy consumption accounts for more than 90% of total energy consumption in Jilin Province, of which coal consumption accounts for more than 65%, which obviously does not meet the development requirements of a low-carbon economy. In order to meet the development needs of emerging industries and the requirements of CO_2_ emission reduction, according to the simulation results, the energy structure adjustment of Jilin Province should be carried out in two steps. The first step is to gradually replace some coal energy consumption with oil and natural gas. However, considering the shortage of fossil energy, the second step is to continuously increase the proportion of renewable energy consumption in order to gradually replace fossil energy consumption.

Taking Jilin Province as an example, this study reveals the correlation between economic growth and CO_2_ emissions under dual structural effects attributed to the Kaya identity of CEI. Owing to scenario analysis, the dynamic impacts of structural effects can be explored. However, the setting of the scenarios is based on the local development status and future planning. It is impossible to ensure the universal applicability for the transformation regions. Although each factor for future development is set as far as possible from the perspective of rationality and feasibility, there are still uncertainties, especially considering the periodicity of policy implementation. We used a five-year interval as a unit of parameter setting, which may induce deviations compared with the actual development situation. In addition, due to the lack of relevant planning, this study did not introduce technological factors into the simulation model, without further exploring the impacts of technological effects on the correlation between economic growth and CO_2_ emissions. In the future, we will further improve our research in the following two respects. The first is to expand the scope of the research and take other transformation regions as targets, so as to make the research more widely representative. The second is to continuously update the scenario settings according to the actual development situation to ensure the accuracy of the prediction results.

## 6. Conclusions

This study aims to unravel the dynamic driving mechanism of dual structural effect on the correlation between economic growth and CO_2_ emissions in a typical transformation region represented by Jilin province. We built an integrated simulation model to cover the interrelationships among the variables, and the prediction of CO_2_ emissions was based on the combination of GDP and CO_2_ emission intensity. The determination of the latter was based on the Kaya identity which is built incorporating the structural factors including industrial structure and energy structure. In order to quantitatively analyze the influence of structural effects on CUG, the concepts of marginal utility and total utility were introduced, and VDA based on the VAR model was employed to quantify the effects. The specific conclusions derived from the present study can be listed as follows:

(1) In terms of the impacts of structural effects on the changing trend in the correlation between GDP and CO_2_ emissions, the slower the adjustment of energy structure and industrial structure is, the more significant the growth trend in GDP will be before the turning point, resulting in significant differences in GDP and CO_2_ emissions at the turning point. The slower decrease in CEI is, the later CO_2_ emissions decouple from GDP.

(2) Lower GDP growth rate and greater decrease in CEI caused by more aggressive adjustment of energy structure and industrial structure results in earlier arrival of the emission peak. The faster GDP grows, the larger CO_2_ emissions, and the later the corresponding turning point appears.

(3) Even under different socio-economic development modes, when CO_2_ emissions peak, the energy structure is different while the industrial structure tends to be similar. Meanwhile, the contribution of the dual structural effects to CUG is basically the same (around 23–24%). The change in industrial structure has a positive driving effect on CUG, while the change in energy structure has a negative driving effect. With the transformation of the socio-economy, the positive driving effect of the industrial structure will gradually weaken, while the negative driving effect of the energy structure will increase.

## Figures and Tables

**Table 1:** The descriptive statistics of the variables.

Variable	Observation	Standard Error	Max	Mean	Min
*CO_2_* (Mt)	21	50.36	222.10	144.76	86.78
*GDP* (billion yuan)	21	456	1406	564	114
*CEI* (t/thousand yuan)	21	20.13	85.54	38.60	14.04
*SI* (%)	21	5.61	53.41	45.04	37.14
*FEI* (tce/thousand yuan)	21	8.18	34.77	15.69	5.71
*CC* (%)	21	9.92	78.70	68.55	53.00
*NG* (%)	21	1.13	3.70	1.94	0.50
*RN* (%)	21	1.04	6.48	5.06	3.23

**Table 2:** Descriptions of the scenarios.

Scenario	Characteristic	Variables			
		*SI*	*RN*	*GDP*	*NG*
S1	Continuation of the historical development trajectory	Continuation of the historical annual change rate of −0.6%	Continuation of the historical annual change rate of 4.3%	Continuation of the historical annual change rate of 6.9% from 2016 to 2020	Annual change rate of 4.8% from 2016 to 2050, reaching 18% in 2050
S2	Reaching an ideal development state as in the future planning.	A certain degree of adjustment at an annual change rate of −1%	A certain degree of adjustment at an annual change rate of 4.8%	Annual change rate of 6.5% from 2016 to 2020	
S3	Basically in sync with the average level of China	In-depth adjustment with an annual change rate of −1.4%	In-depth adjustment with an annual change rate of 5.2%	Annual change rate of 6.2% from 2016 to 2020	
S4	Basically reaching the level of the developed countries	More in-depth adjustment with an annual change rate of −1.8%, lower than 30% in 2050	More in-depth adjustment with an annual change rate of 5.6%, more than 40% in 2050	Annual change rate of 6.0% from 2016 to 2020	

**Table 3:** Settings for variables in four scenarios during 2016–2050.

Variable	Scenario	2020	2025	2030	2035	2040	2045	2050
*SI* (%)	S1	48.34	46.91	45.52	44.17	42.86	41.59	40.36
	S2	47.38	45.06	42.85	40.75	38.75	36.85	35.05
	S3	46.43	43.27	40.32	37.58	35.02	32.64	30.42
	S4	45.49	41.54	37.94	34.64	31.64	28.89	26.38
*RN* (%)	S1	8.50	11.37	15.14	19.79	23.62	26.46	27.95
	S2	8.91	12.79	17.93	23.77	28.10	31.48	33.58
	S3	9.29	13.84	20.05	26.21	31.58	36.08	38.87
	S4	9.73	15.25	22.40	29.98	36.47	41.26	44.45
*GDP* (billion yuan)	S1	1963	2677	3566	4638	5892	7203	8679
	S2	1927	2578	3370	4301	5360	6521	7745
	S3	1900	2518	3260	4122	5087	6130	7211
	S4	1882	2495	3184	3970	4828	5734	6647
*NG* (%)	S1–S4	4.42	5.59	7.07	8.94	11.30	14.29	18.06
*CEI* (t/thousand yuan)	S1	11.84	9.76	8.05	6.63	5.47	4.51	3.71
	S2	11.45	9.13	7.28	5.81	4.63	3.69	2.94
	S3	11.11	8.60	6.65	5.15	3.98	3.08	2.38
	S4	10.78	8.09	6.07	4.56	3.42	2.57	1.93

**Table 4:** Parameter estimation results for Equations (4)–(6).

Parameter	Coefficient	T-statistics	Probability	Adjusted R^2^
*a*	0.9121 ***	3.7908	0.0016	0.9625
*b*	0.3387 ***	1.2684	0.0000	
*c*	0.8670 ***	3.5843	0.0026	
*d*	−0.1494 ***	−2.9451	0.0044	
*e*	−6.5402 ***	−22.8653	0.0000	
*m*	−0.9302 ***	−12.9450	0.0000	0.9998
*δ_1_*	1.2181 ***	271.8653	0.0000	
*n*	−0.3525 ***	−4.4893	0.0002	0.9356
*δ_2_*	4.9235 ***	19.5926	0.0000	

Note: *** indicates significance at the 1% level.

**Table 5:** Parameter estimation results for Equation (1).

Variable	Scenario			
	S1	S2	S3	S4
ln*GDP*	3.56 ***	4.00 ***	4.40 ***	4.79 ***
ln*GDP*^2^	−0.16 ***	−0.19 ***	−0.21 ***	−0.24 ***
*λ*	−9.47 ***	−11.16 ***	−12.70 ***	−14.24 ***
Adjusted R^2^	0.9968	0.9808	0.9803	0.9943

Note: *** indicates significance at the 1% level.

**Table 6:** Values of the variables at the turning point in four scenarios.

Variable	Scenario			
	S1	S2	S3	S4
*CEI* (t/10^3^ yuan)	4.51	5.81	6.65	8.09
*NG* (%)	14.29	8.94	7.07	5.59
*RN* (%)	22.98	16.60	13.90	11.21
*SI* (%)	41.59	40.75	40.32	41.54
*CC* (%)	45.73	51.25	54.53	58.80
*FEI* (tce/10^3^ yuan)	1.84	2.48	2.93	3.58

**Table 7:** Contributions of structural effects (*CES* and *CIS*) to CUG (%).

Year	CIS				CES			
	S1	S2	S3	S4	S1	S2	S3	S4
2016–2020	38.44	37.43	36.67	35.64	−5.56	−6.48	−7.69	−8.56
2021–2025	37.21	35.85	34.37	33.01	−6.11	−6.99	−8.25	−9.38
2026–2030	36.28	34.21	31.98	31.18	−6.44	−7.74	−8.87	−10.24
2031–2035	35.13	32.97	30.92	28.77	−7.07	−8.16	−9.73	−11.24
2036–2040	33.24	31.33	29.03	27.01	−7.86	−9.48	−10.57	−12.49
2041–2045	32.45	30.15	28.01	26.12	−8.66	−10.06	−11.96	−13.45
2046–2050	30.46	29.03	27.31	25.38	−9.34	−11.01	−12.93	−14.32
Average	32.45	32.97	31.98	33.01	−8.66	−8.16	−8.87	−9.38

As the stability and co-integration of the time series variables have been verified, the VAR model was constructed directly and a stationarity test was performed for the model. The inverse roots of the AR characteristic polynomial in the four scenarios were all less than 1, indicating that the VAR model is a stationary system and the VDA could be conducted on this basis.

## Data Availability

The data presented in this study are available upon request from the corresponding author.

## Abbreviations

**Abbreviation**	**Term**
CEI	CO_2_ emission intensity
VDA	Variance decomposition analysis
CIS	The change in industrial structure
CES	The change in energy structure
CUG	CO_2_ emissions caused by unit GDP growth
MU	Marginal utility
TU	Total utility
RN	The proportion of renewable energy
